# Community-Based Seroprevalence of SARS-CoV-2 Antibodies following the First Wave of the COVID-19 Pandemic in Jazan Province, Saudi Arabia

**DOI:** 10.3390/ijerph182312451

**Published:** 2021-11-26

**Authors:** Abdulaziz Alhazmi, Edrous Alamer, Siddig Abdelwahab, Nizar Khamjan, Abdullah Hamami, Moayad Haddad, Majid Darraj, Anwar M. Hashem, Abdullah Algaissi

**Affiliations:** 1 Microbiology and Parasitology Department, Faculty of Medicine, Jazan University, Jazan 45142, Saudi Arabia; abalhazmi@jazanu.edu.sa; 2 Emerging and Epidemic Infectious Diseases Research Unit, Medical Research Center, Jazan University, Jazan 45142, Saudi Arabia; ealamer@jazanu.edu.sa (E.A.); sadiqa@jazanu.edu.sa (S.A.); 3 Department of Medical Laboratories Technology, College of Applied Medical Sciences, Jazan University, Jazan 45142, Saudi Arabia; nkhamjan@jazanu.edu.sa; 4 Prince Mohammed Bin Nasser Hospital, Ministry of Health, Jazan 45142, Saudi Arabia; ah7mmami@gmail.com; 5 King Fahad Central Hospital, Ministry of Health, Jazan 45142, Saudi Arabia; m.hedad@gmail.com; 6 Medicine Department, Faculty of Medicine, Jazan University, Jazan 45142, Saudi Arabia; mdarraj@jazanu.edu.sa; 7 Vaccines and Immunotherapy Unit, King Fahd Medical Research Center, King Abdulaziz University, Jeddah 21859, Saudi Arabia; amhashem@kau.edu.sa; 8 Department of Medical Microbiology and Parasitology, Faculty of Medicine, King Abdulaziz University, Jeddah 21859, Saudi Arabia

**Keywords:** COVID-19, SARS-CoV-2, seroprevalence, anti-S antibodies, ELISA

## Abstract

Severe acute respiratory syndrome coronavirus 2 (SARS-CoV-2) continues to spread globally, causing unprecedented effects on global health and economies. Community-based serological data are essential for understanding the true prevalence of infections, specifically the subclinical infections, as COVID-19 asymptomatic infections are common. Such data would also be important for decision making around choosing appropriate epidemiological control measures, as well as for the true estimation of mortality rates in the population. Further, determining the seroprevalence of anti-SARS-CoV-2 antibodies in the population would provide important information on herd immunity. In this study, we conducted a population-based age-stratified serological study to understand the prevalence of SARS-CoV-2 in Jazan Province, Saudi Arabia. Out of 594 participants who were recruited from 29 August to 30 December 2020, just before the vaccination rollout program in Saudi Arabia, about 157 were seropositive for SARS-CoV-2, indicating an estimated seropositivity rate of 26%. Although no significant difference in seropositivity was seen between male and female participants, we found that lower seroprevalence was associated with the younger (below 18 years old) and older populations (older than 56 years) compared with other age groups (19–55 years). These data indicate a high prevalence of SARS-CoV-2 antibodies following the peak of COVID-19 spread in Jazan province; however, most of the population (three-quarters) remains susceptible to SARS-CoV-2 infection.

## 1. Introduction

Since its emergence in late December 2019, severe acute respiratory syndrome coronavirus 2 (SARS-CoV-2), which caused the COVID-19 pandemic, has spread to more than 200 countries and territories around the world, causing an enormous impact on global public health [1]. In Saudi Arabia, confirmed cases have been detected on a daily basis since early March of 2020 and continue to be detected now. As of July 2021, about 521,239 confirmed COVID-19 cases were identified in Saudi Arabia, with about a 1.57% fatality rate, according to the Saudi ministry of health (MOH) daily reports [2]. Jazan province, with its 13 cities, has reported more than 21,000 cumulative confirmed cases of COVID-19 thus far, which makes it one of the provinces with the highest number of COVID-19 cases; it has the fifth highest number out of the 13 provinces in regard to the total reported cases in the kingdom.

The SARS-CoV-2 is a novel coronavirus (CoV) that is classified within the subgenus Sarbecovirus of the genus Betacoronavirus, along with SARS-CoV-1 and several emerging animal CoVs. Its genome is approximately 30 kb and contains at least six open reading frames (ORFs) [3]. The first two-thirds of the genome length codes for polyproteins: pp1a and pp1ab that are processed by viral and host proteases into 16 non-structural proteins (nsps) [4], while the other third of the genome encodes the four main structural proteins (envelope (E), membrane (M), spike (S), and nucleocapsid (N) proteins) and other accessory proteins [4]. The S protein is the main protein for attachment and entry to host cells and consists of two subunits: S1, which contains the receptor-binding domain (RBD) that mediates the virus’ binding to cellular receptors, and S2, which mediates fusion with cell membranes. Most of the antibodies against SARS-CoV-2 infection are generated against the S protein. Hence, almost all currently approved vaccines target the S as an immunogen [5]. For this same reason, the detection of antibodies against the S protein has been the major focus of serological assays. Another important protein for SARS-CoV-2 serological assays is the N protein [6].

One of the characteristics of COVID-19 infection is the prevalence of asymptomatic infections which appear to contribute to the silent community transmission of the infection [7,8,9,10,11]. Asymptomatic transmission makes it difficult to trace and isolate infected cases early on [7,8,9,10,11]. To date, case detection has focused primarily on patients with symptoms or a severe form of the disease, because most of the patients with mild or asymptomatic infections do not know if they have been infected and, thus, do not seek medical attention [12]. Serological studies are critically important to discover information about the true extent of the spread of the virus in the community by revealing these mild and asymptomatic infections. In addition, community serological data are essential for understanding the percentage of the population’s herd immunity against SARS-CoV-2, which is critical information for implementing vaccines. Population serological data would also be important for decision making on epidemiological control measures and are an essential factor in eliminating the infection [13,14,15]. In Saudi Arabia, which experienced the peak of the first wave of the COVID-19 pandemic between May and July 2020, there has been a very limited number of community-based and blood donor-based serological studies on the national and local levels, with seroprevalence rates ranging from 1.6% to 19% [16,17,18,19,20,21]. This variation is expected due to the different points in time when studies were conducted (summarized in Table 1). In this study, we aimed to conduct a population-based age-stratified serological study to understand the prevalence of SARS-CoV-2 in Jazan Province. This study presents estimated seroprevalence based on a commercial ELISA and a validated in-house ELISA to detect specific IgG antibodies against SARS-CoV-2 S1 and N antigens.

## 2. Materials and Methods

### 2.1. Study Settings and Subjects 

In this prospective cohort study, volunteers from Jazan province were randomly recruited to determine the prevalence of SARS-CoV-2-specific immunoglobulin G (IgG) antibodies. Participants were recruited on a voluntary basis to visit the Jazan University Hospital if they agreed to participate in the study. Individuals from all age groups were included and gave informed consent to information that was provided to all participants, or their legal guardians, before they were enrolled in the study (Table 2). Questions about demographic data (such as age, sex, health, social status, and region) were asked electronically as a health precautionary measure, and different verification steps were taken by the study members. Participants who refused to participate, pregnant women, and patients with known chronic immunological diseases were excluded from this study. Blood samples were obtained from 671 recruited individuals; however, only 594 samples were used in the study as 77 samples were excluded because their data were missing. The period of recruitment of participants and sample collection was between the 25th of August and 30th of December 2020. It is worth mentioning that recruitment and sample collection were finished before the beginning of COVID-19 vaccination in Saudi Arabia, which begin in January 2021; thus, none of the participants had received any of the COVID-19 vaccines at the time of the study.

### 2.2. Determination of Anti-SARS-CoV-2 IgG Antibodies by ELISA

After collecting blood samples from all participants, sera were separated by centrifugation and stored at −20 °C until they were used for assays. To determine the seroprevalence of SARS-CoV-2 IgG antibodies, we utilized indirect enzyme-linked immunosorbent assays (ELISA) kits purchased from Vircell Microbiologists (Granada, Spain). The tests were performed, interpreted, and validated according to the manufacturer’s instructions. As a confirmatory assay, samples that tested positive with the commercial kits were tested using an in-house ELISA assay that we recently developed and validated [6] to measure SARS-CoV-2 IgG antibodies against spike glycoprotein (S). Briefly, commercial recombinant SARS-CoV-2 S1 subunit (amino acids 1–685) (Sino Biological, China) and an in-house-produced recombinant SARS-CoV-2 N protein were used to coat 96-well ELISA plates at 1 μg/mL and 4 μg/mL in phosphate-buffered saline (PBS), respectively, overnight at 4 °C. Plates were then washed with PBS with 0.05% Tween-20 (PBS-T). After blocking the plates with 5% skim milk in PBS-T buffer at 37 °C for one hour, 1:100 diluted serum samples were incubated for one hour at 37 °C and washed. Anti-IgG HRP-conjugated antibodies (Jackson ImmunoResearch, West Grove, PA, USA) were then added for one hour at 37 °C. Following incubation, plates were washed and 3,3′,5,5′-tetramethylbenzidine (TMB) substrate (KPL, Gaithersburg, MD, USA) was added for 30 minutes in the dark at room temperature, and the reaction was terminated by 0.16 M sulfuric acid. Absorbance was measured at 450 nm using an ELx808 microplate reader (BioTek, Winooski, VT, USA). Cutoff values for the in-house ELISA were 0.4 for N-ELISA and 0.17 for S1-ELISA, as previously determined [6]. Serum samples collected pre-COVID-19 pandemic (n = 9) were used in both ELISA assays as a negative control.

### 2.3. Ethical Statement

This study was approved by the Research Ethics Committee of Jazan University, Saudi Arabia (IRB Approval number REC42/1/001, approved date 19 August 2020). All participants in the study signed informed consent forms before providing blood samples.

### 2.4. Statistical Analyses

The outcome variable was SARS-CoV-2 seropositivity, defined as an OD450 value of 0.30 or higher using the in-house anti-S1 ELISA, while the independent variables included gender and age group. Age groups were divided into the following categories: less than 18 years, 19–30 years, 31–55 years, and older than 56 years old. The seroprevalence of SARS-CoV-2 antibodies was calculated with 95% CI as proportions. Statistical significance for both comparisons was assessed using the Chi-square test or Fisher’s exact test for categorical variables, and the T test for quantitative continuous variables. Statistical analyses were conducted using GraphPad Prism 9 software and SPSS v.23.

## 3. Results

### 3.1. Characteristics of Study Participants

The median age of the 594 participants was 30 ± 9.69 years with a range of 1 to 75. The study population included 362 (61%) male and 231 (39%) female participants. Among the participants, 33 (5.1%) reported that they had been diagnosed with COVID-19 in the past and were included in the analysis, while 26 (4.1%) reported experiencing COVID-19-related symptoms without SARS-CoV-2 PCR confirmation. Furthermore, 31 participants (5.1%) reported having chronic health issues (Table 2).

### 3.2. Seroprevalence of SARS-CoV-2 Antibodies in Study Participants

Seropositivity in this study was determined using a commercial ELISA kit purchased from Vircell Microbiologists (REF # G1032, Granada, Spain) and an in-house ELISA assay that was previously developed and validated by our group [6]. To obtain the maximum sensitivity, samples that tested positive with the commercial ELISA kit were subjected to confirmatory testing using our in-house indirect ELISA to detect IgG antibodies against SARS-CoV-2 S1 and N proteins in serum samples [6]. Since most of the binding antibodies are generated against the Spike protein (S), which contains within its subunit 1 (S1) the receptor-binding domain (RBD) that facilitates binding to the host cell receptor and against which the majority of antibodies are generated, we only considered samples that were positive for S1 IgG as confirmed positives, as previously described [6,21]. Using Vircell ELISA kits led to the identification of 157/594 SARS-CoV-2 seropositive cases. We then used our in-house N- and S1-based ELISA assays to confirm the seropositivity. Thus, samples that were positive with the Vircell ELISA (157 samples) were tested using the in-house N and S1 ELISA. Our data show that 157/157 and 153/157 samples were seropositive for SARS-CoV-2 N and S1, respectively. To obtain the maximum sensitivity, we only considered samples that tested positive by three assays as positive sample and used them to estimate the seropositivity of our analytic sample, which was found to be 25.75% (153/594) with a CI of 21.2–30.0 (Table 3). Among all 153 seropositive cases, 88 were male (57.51%; 49.3–64.3), while 65 were female (42.49%; 38.2–48.6). In addition, the seropositivity of SARS-CoV-2 in our population varied among the different age groups with a rate of 25%, 32%, 30.8%, and 10.1% for 1 to 18 years, 19 to 30 years, 31 to 55, and 56 to 75 years, respectively (Table 4).

## 4. Discussion

COVID-19 is a pandemic disease caused by SARS-CoV-2. The first case of COVID-19 was reported in Saudi Arabia on the 2nd of March 2020. Fever, cough, fatigue, loss of smell and taste, and shortness of breath are the most prevalent symptoms associated with COVID-19 [22,23,24,25]. There are different methods for SARS-CoV-2 detection. Real-time Polymerase Chain Reaction (RT-PCR) is considered the most reliable technique for COVID-19 diagnosis due to its higher sensitivity and specificity. Serological assays, such as ELISA, are reliable in detecting immune responses against SARS-CoV-2, and have been widely applied in seroprevalence studies [26]. The benefits of seroprevalence studies include identifying the fraction of asymptomatic people and the incomplete ascertainment of people with symptoms among the general population, which is important for decision making around choosing appropriate epidemiological control measures, as well as for the true estimation of mortality rates in the population. Furthermore, determining the seroprevalence of anti-SARS-CoV-2 antibodies in the population would provide important information on herd immunity. These studies are strongly recommended by WHO to evaluate the exposure of the general population to this novel virus [27].

**Figure 1:**
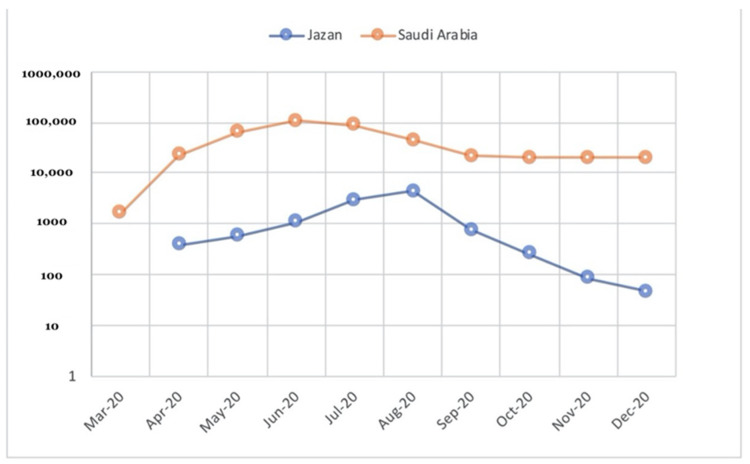
Cumulative monthly confirmed COVID-19 cases in Jazan province and Saudi Arabia, March–December 2020. (Data source: Saudi ministry of health, COVID-19 portal.)

Since the first case reported in the country on 2nd March 2020, Saudi Arabia has taken strict measures on national and international levels to control SARS-CoV-2 transmission and its consequences. These measures included a travel ban, closure of holy places, schools, and universities, and ended with the total lockdown of the country [2]. Despite these measures, COVID-19 cases have risen to reach their peak in June and July 2020 (Figure 1). All cities in Saudi Arabia have been affected by COVID-19 with considerable variations in the daily cases, mortality rates, and hospitalizations. Despite its early arrival to Jazan, a south-western province in Saudi Arabia, SARS-CoV-2 reached its peak late compared to bigger provinces such as Riyadh, Jeddah, and the eastern provinces. To date, only a small number of serological studies have been conducted in Saudi Arabia and none in Jazan province (summarized in Table 1). Furthermore, most of the studies were conducted early in the COVID-19 pandemic in Saudi Arabia and targeted specific populations such as health care workers and blood donors; thus, there is a need for serological studies to be conducted in Saudi Arabia, particularly community-based studies [17,18,19,20,21].

In this study, samples were collected between the 25th of August and 30th of December 2020, just a few weeks after the peak of the first wave of the COVID-19 pandemic in Jazan province and just before the massive vaccination campaigns that were initiated by national health officials in Saudi Arabia (Figure 1). We randomly targeted the general population of the community regardless of their age, sex, job status, or COVID-19 status. The overall seroprevalence in our study is about 25.75%. This percentage is higher than the results of all of the population-based seroprevalence studies that were conducted in Saudi Arabia (Table 1), which can be explained by the time of our study and the target population. It is noteworthy that the rates of seroprevalence are increasing, if the dates of these studies are considered, regardless of the included population, from 0% to about 30% (Table 1), and these patterns are largely consistent with the epidemiological curve of COVID-19 cases in Saudi Arabia (Figure 1).

Our study has a few limitations. For example, because of the lack of accessibility to biosafety level 3 (BSL3), we were unable to conduct the neutralizing assays to detect the presence of neutralizing antibodies in our cohort. However, previous studies have shown a strong correlation between the levels of binding antibodies (i.e., IgG Abs) and the levels of neutralizing in sera derived from COVID-19 patients as well as recovered individuals [28]. In addition, we reported only IgG isotype antibodies in this study. Testing for viral RNA by PCR or detection of IgM antibodies would be beneficial to detect cases at the early stages of infection. Nevertheless, in our study we used multiple assays to capture as many positive cases as possible and to obtain maximum sensitivity. For instance, we used a commercial ELISA assay with a validated high sensitivity and specificity and followed that with a validated and well-characterized in-house ELISA in which positive samples were tested for antibodies against N antigen and S1. These three-step confirmatory assays were performed to capture as many potential SARS-CoV-2-positive cases as possible. Additionally, as S1 mediates binding and entry into cells and being a target for neutralizing antibodies, the S protein is under continuous selective pressure, which makes it more prone to acquiring mutations that might affect the accuracy of S-based serological assays. Therefore, we believed that inclusion of both S1 and N in serological testing may have overcome the aforementioned issues

In our study, the seroprevalence status of SARS-CoV-2 was compared among different subpopulations. Neither sex nor age is associated significantly with higher seroprevalence. Similar to different international population studies, no sex difference was reported in COVID-19 seropositivity; however, our data finds a trend of higher seroprevalence in the adult population (31–55 years old) compared with children and older people [29,30]. Moreover, in Saudi Arabia, Banjar et al. found that there is no significant difference among different age groups in COVID-19 seropositivity in blood donors, which is consistent with our findings. Furthermore, the high prevalence rate found in this study might indicate a high silent (asymptomatic) transmission rate, which suggests a need to increase testing for COVID-19 in the community so that positive asymptomatic cases can be picked up early on before they transmit the infection to others.

Taken together, our community-based seroprevalence study revealed a high prevalence of SARS-CoV-2 antibodies in the population following the peak of the COVID-19 pandemic in Jazan province, and that the adult population might have a higher seropositivity rate compared to children and elderly populations, suggesting higher exposure rates for this age group to SARS-CoV-2 infection. This study has important implications around the spread of SARS-CoV-2 in the Jazan region and the effectiveness of applied public health measures. The data could also have implications for the prioritization of COVID-19 vaccinations.

## Figures and Tables

**Table 1:** Summary of different seroprevalence studies in Saudi Arabia.

Scheme	Period of Study	Technique	Seroprevalence %	Population (n)	City
Alandijany et al. (13)	01.01.2020 to 31.05.2020	ELISA CLIA MN	0	Blood donors (956)	Jeddah
Banjar et al. (14)	20.05.2020 to 25.05.2020	CLIA	1.4	Blood donors (837)	Nationwide
Alserehi et al. (15)	20.05.2020 to 30.05.2020	CMIA	2.9	Health-care workers (9379)	Nationwide
Mahallawi et al. (16)	15.05.2020. to 15.06.2020	ELISA	19.3	Blood donors (1212)	Al-Madinah
Ahmed et al. (17)	01.06.2020 to 01.07.2020	ELISA MN	6.3	HCW (204)	Makkah
Alhabbab et al. (18)	29.06.2020 to 10.08.2020	ELISA MN	32.2	HCW, quarantine sites (693)	Jeddah
Current study	25.08.2020 to 30.12.2020	ELISA	26	General population (594)	Jazan

ELISA: enzyme-linked immunosorbent assay. CLIA: chemiluminescence immunoassay. MN: microneutralization assay. CMIA: chemiluminescent microparticle immunoassay. HCW: healthcare workers.

**Table 2:** Characteristics of study participants.

Characteristic	Category	Number of Participants n (%)
Overall		594 (100%)
Gender	Male	362 (61%)
	Female	231 (39%)
Age ^a^		30 ± 9.69
Age groups	<18	108 (18.01%)
	19–30	177 (27.79%)
	31–55	211 (35.52%)
	>56	98 (16.49%)
Confirmed previous COVID-19	Yes	33 (5.1%)
	No	561 (94.6%)
Reported any COVID-19-related symptoms	Yes No	26 (4.1%) 568 (95.9%)
Presence of chronic health issues ^b^	Yes	31 (5.2%)
	No	563 (94.8%)

^a^ Mean ± SD, ^b^ Reported chronic health issues to include diabetes, hypertension, chronic kidney diseases, asthma, and chronic hepatitis B infection.

**Table 3:** Number and percentage of seropositive cases in the study population.

	n of Positive/Total (%; 95% CI) ^a^		
Assay	Vircell ELISA kit	In-house ELISA	
		Anti-N IgG	Anti-S1 IgG
Total samples= 594	157/594 (26%)	157/157 (100%)	153/157 (98.08%)
Overall seropositivity in the population	153/594 (25.75%; 21.2–30.0)		

^a^ 95% confidence intervals.

**Table 4:** Seropositivity by gender and age groups.

		Total (n)	Seropositivity n (%; 95% CI)
	Overall	594	153 (25.75%; 21.2–30.0)
Gender	Male Female	362	88 (57.51%; 49.3–64.3) 65 (42.49 %; 38.2–48.6)
		232	
Age group (years)	<18 19–30 31–55 >56	108 177 211 98	41 (27.1%; 20.1–30.8) 50 (32.0%; 46.6–55.1) 47 (30.8%; 41.3–54.9) 15 (10.1.0%; 4.1–14.9)

## Data Availability

The data presented in this study are available on request from the corresponding author.
